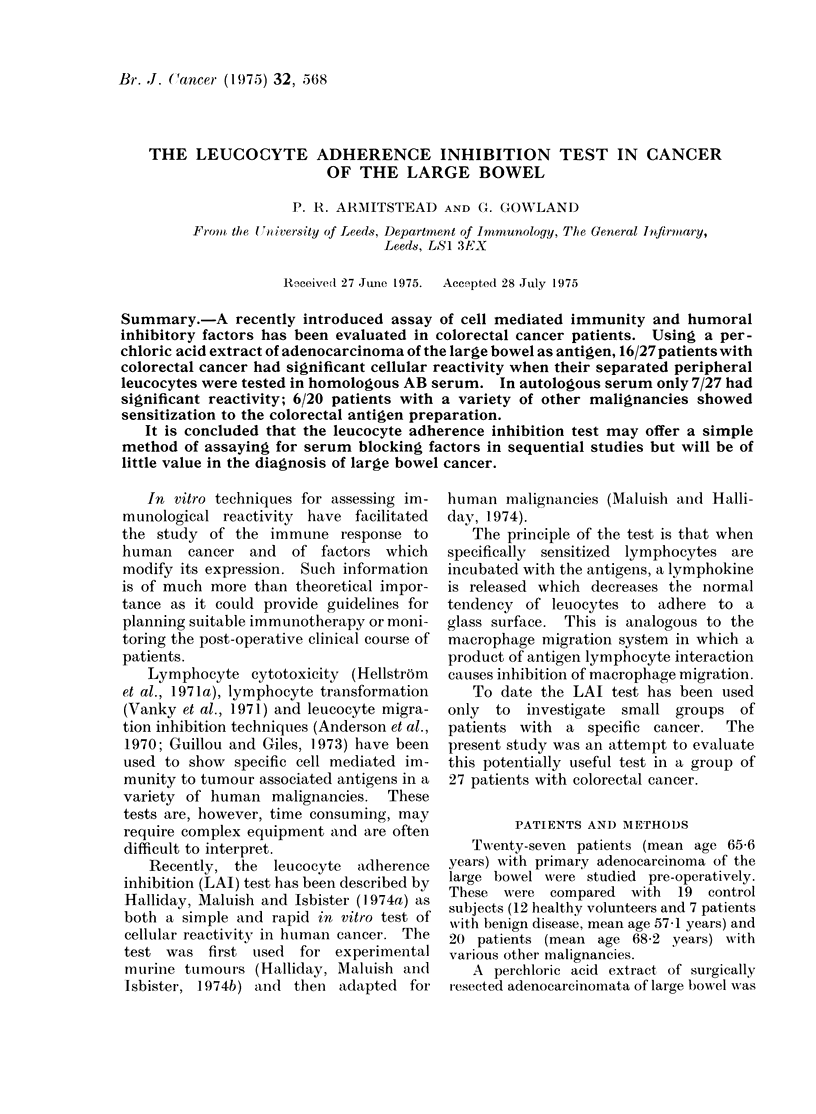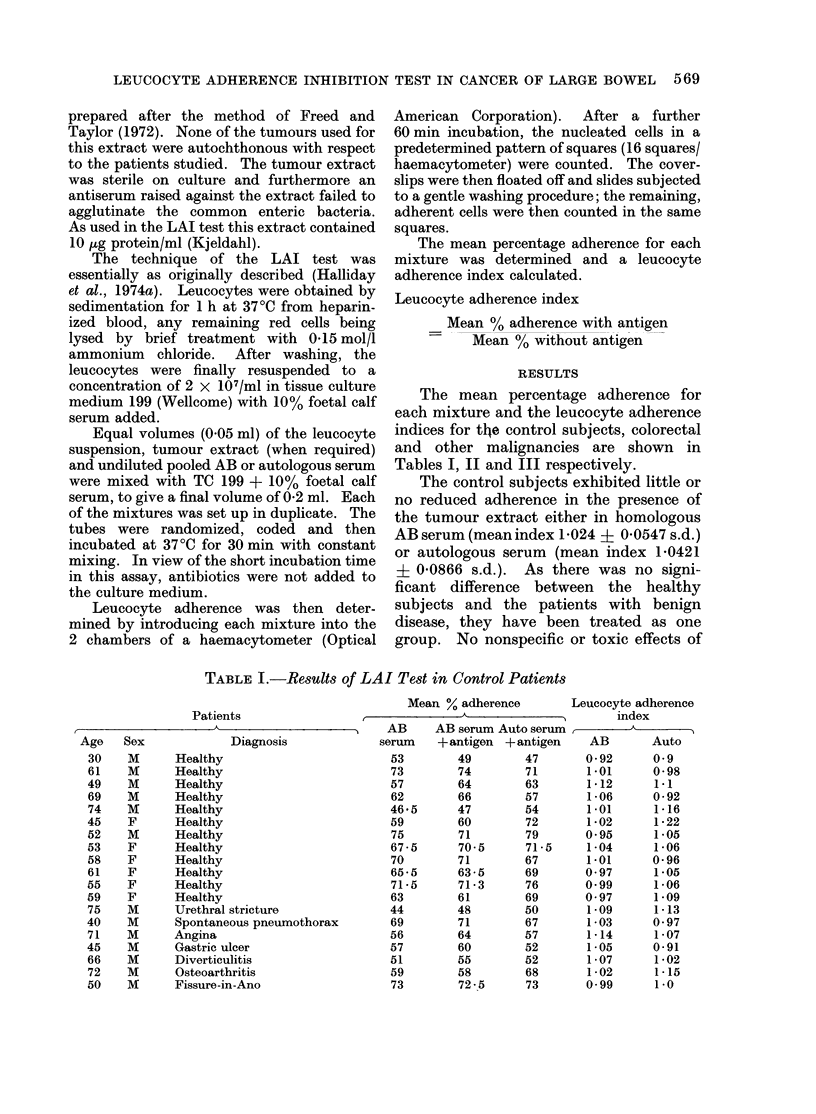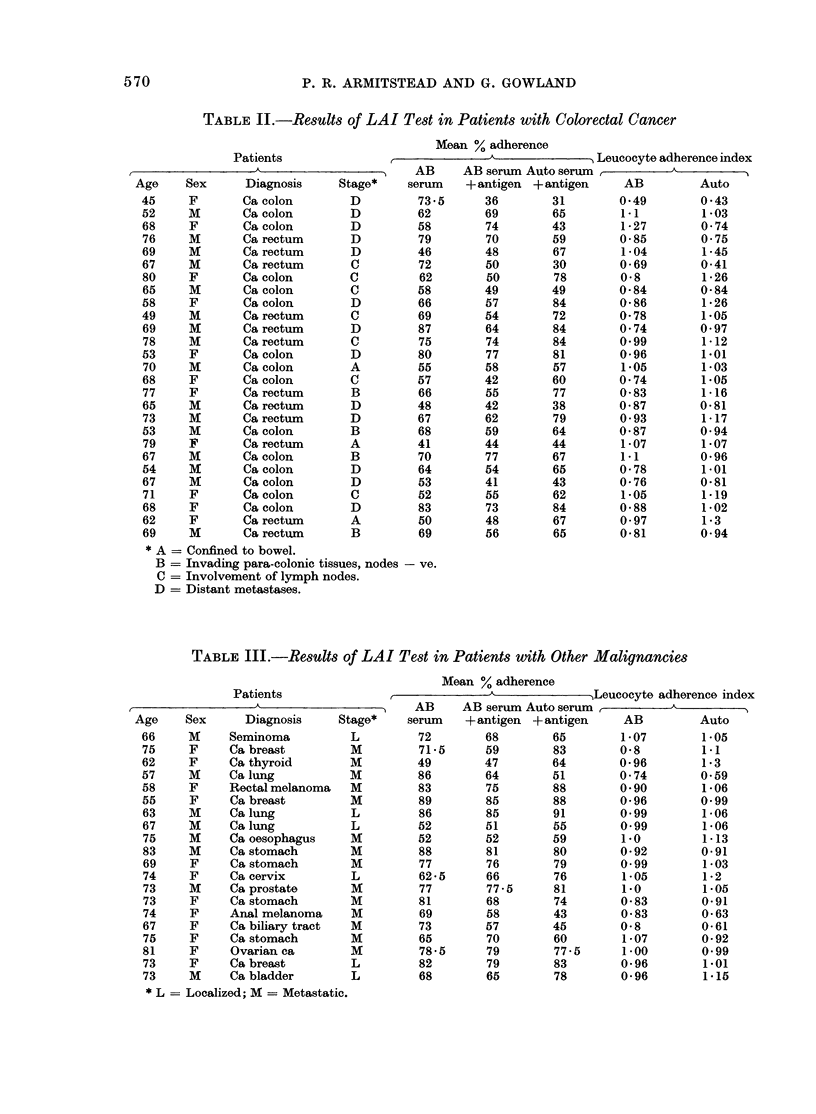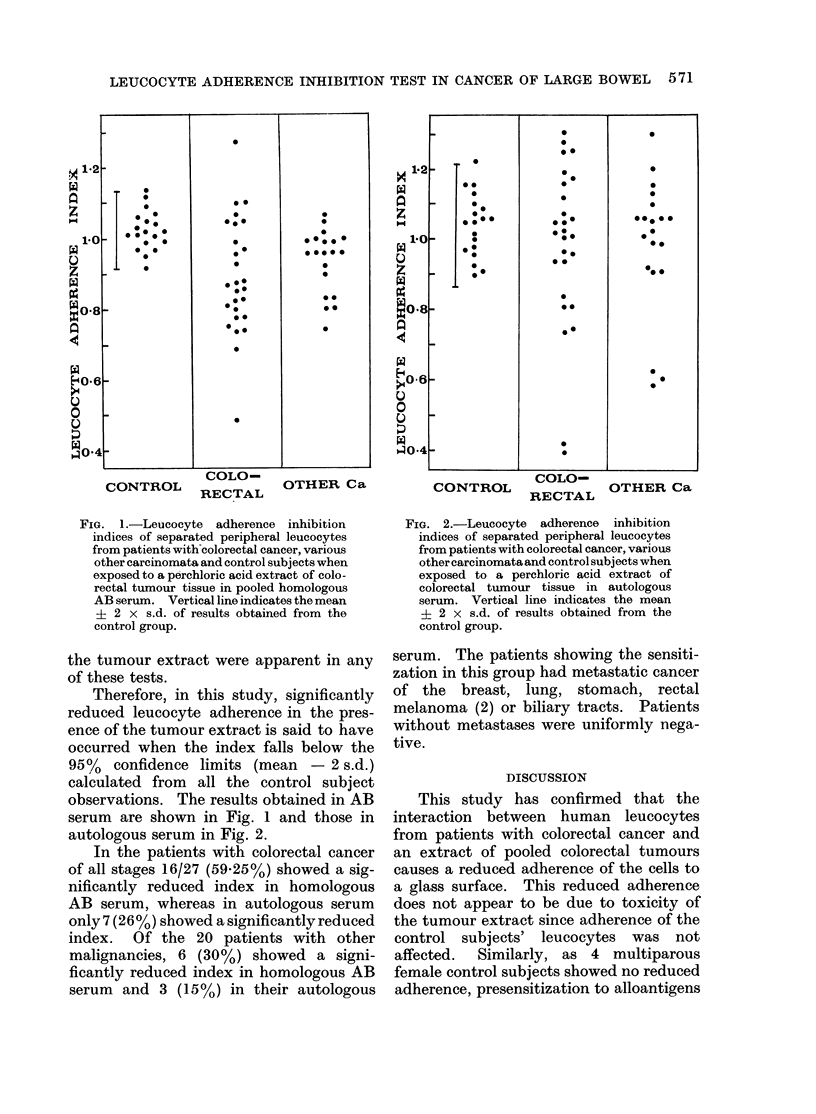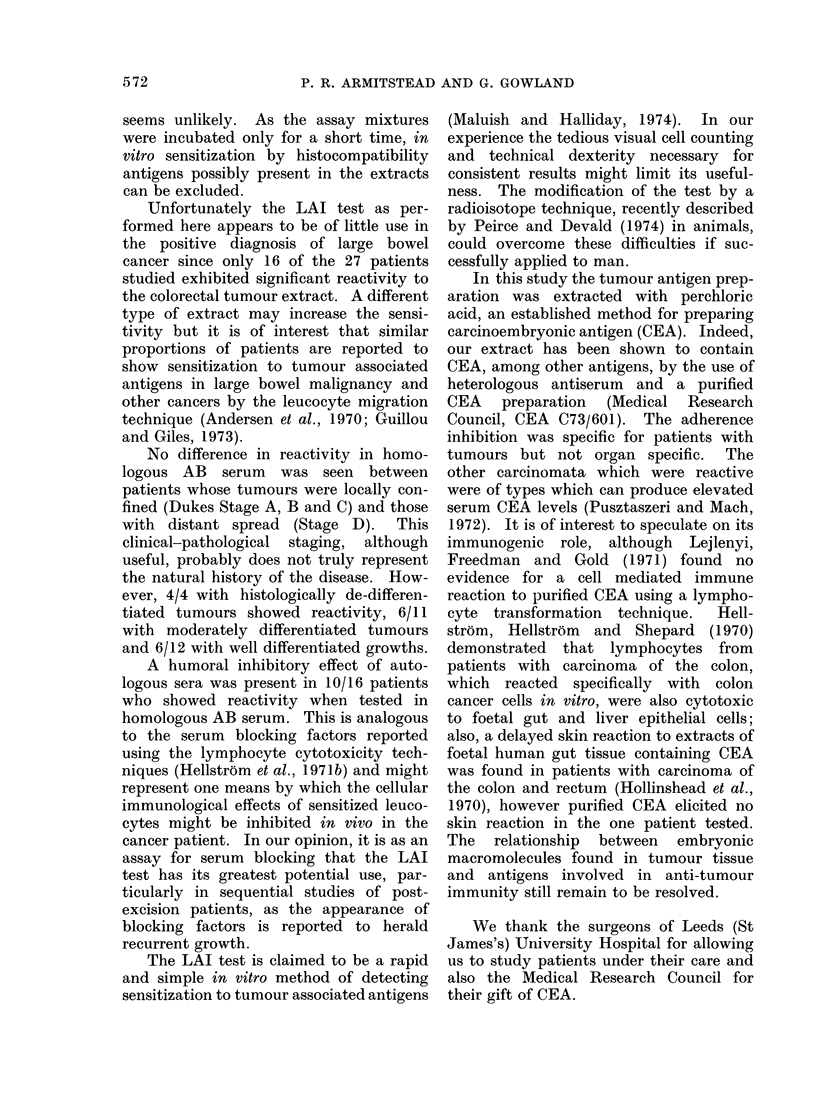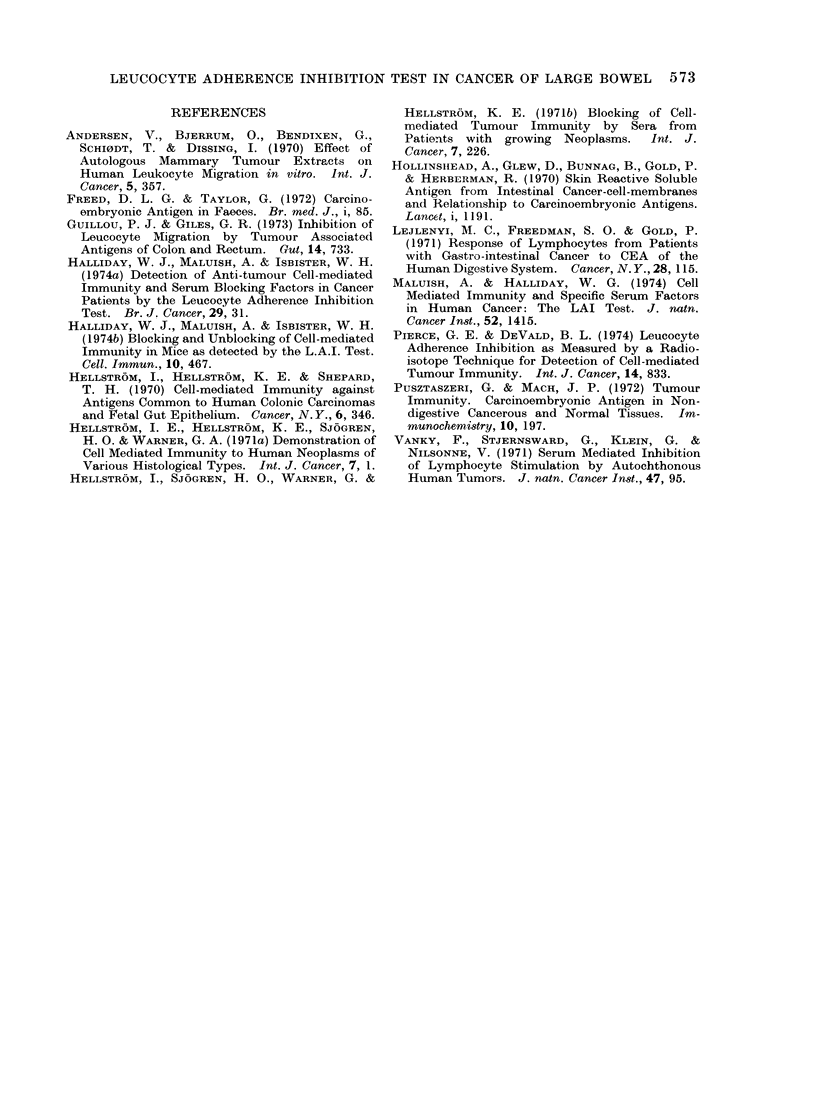# The leucocyte adherence inhibtion test in cancer of the large bowel.

**DOI:** 10.1038/bjc.1975.262

**Published:** 1975-11

**Authors:** P. R. Armitstead, G. Gowland

## Abstract

A recently introduced assay of cell mediated immunity and humoral inhibitory factors has been evaluated in colorectal cancer patients. Using a perchloric acid extract of adenocarcinoma of the large bowel as antigen, 16/27 patients with colorectal cancer had significant cellular reactivity when their separated peripheral leucocytes were tested in homologous AB serum. In autologous serum only 7/27 had significant reactivity; 6/20 patients with a variety of other malignancies showed sensitization to the colorectal antigen preparation. It is concluded that the leucocyte adherence inhibiton test may offer a simple method of assaying for serum blocking factors in sequential studies but will be of little value in the diagnosis of large bowel cancer.


Br. J. Cancer (1975) **32**, 568

### THE LEUCOCYTE ADHERENCE INHIBITION TEST IN CANCER OF THE LARGE BOWEL

P. R. ARMITSTEAD AND G. GOWLAND

*From the University of Leeds, Department of Immunology, The General Infirmary, Leeds, LS1 3EX*

Received 27 June 1975. Accepted 28 July 1975

**Summary.—A recently introduced assay of cell mediated immunity and humoral inhibitory factors has been evaluated in colorectal cancer patients. Using a perchloric acid extract of adenocarcinoma of the large bowel as antigen, 16/27 patients with colorectal cancer had significant cellular reactivity when their separated peripheral leucocytes were tested in homologous AB serum. In autologous serum only 7/27 had significant reactivity; 6/20 patients with a variety of other malignancies showed sensitization to the colorectal antigen preparation.**

**It is concluded that the leucocyte adherence inhibition test may offer a simple method of assaying for serum blocking factors in sequential studies but will be of little value in the diagnosis of large bowel cancer.**

*In vitro* techniques for assessing immunological reactivity have facilitated the study of the immune response to human cancer and of factors which modify its expression. Such information is of much more than theoretical importance as it could provide guidelines for planning suitable immunotherapy or monitoring the post-operative clinical course of patients.

Lymphocyte cytotoxicity (Hellström *et al.*, 1971*a*), lymphocyte transformation (Vanky *et al.*, 1971) and leucocyte migration inhibition techniques (Anderson *et al.*, 1970; Guillou and Giles, 1973) have been used to show specific cell mediated immunity to tumour associated antigens in a variety of human malignancies. These tests are, however, time consuming, may require complex equipment and are often difficult to interpret.

Recently, the leucocyte adherence inhibition (LAI) test has been described by Halliday, Maluish and Isbister (1974*a*) as both a simple and rapid *in vitro* test of cellular reactivity in human cancer. The test was first used for experimental murine tumours (Halliday, Maluish and Isbister, 1974*b*) and then adapted for human malignancies (Maluish and Halliday, 1974).

The principle of the test is that when specifically sensitized lymphocytes are incubated with the antigens, a lymphokine is released which decreases the normal tendency of leuocytes to adhere to a glass surface. This is analogous to the macrophage migration system in which a product of antigen lymphocyte interaction causes inhibition of macrophage migration.

To date the LAI test has been used only to investigate small groups of patients with a specific cancer. The present study was an attempt to evaluate this potentially useful test in a group of 27 patients with colorectal cancer.

### PATIENTS AND METHODS

Twenty-seven patients (mean age 65·6 years) with primary adenocarcinoma of the large bowel were studied pre-operatively. These were compared with 19 control subjects (12 healthy volunteers and 7 patients with benign disease, mean age 57·1 years) and 20 patients (mean age 68·2 years) with various other malignancies.

A perchloric acid extract of surgically resected adenocarcinomata of large bowel was prepared after the method of Freed and Taylor (1972). None of the tumours used for this extract were autochthonous with respect to the patients studied. The tumour extract was sterile on culture and furthermore an antiserum raised against the extract failed to agglutinate the common enteric bacteria. As used in the LAI test this extract contained 10 $\mu$g protein/ml (Kjeldahl).

The technique of the LAI test was essentially as originally described (Halliday *et al.*, 1974*a*). Leucocytes were obtained by sedimentation for 1 h at 37°C from heparinized blood, any remaining red cells being lysed by brief treatment with 0·15 mol/l ammonium chloride. After washing, the leucocytes were finally resuspended to a concentration of $2 \times 10^7$/ml in tissue culture medium 199 (Wellcome) with 10% foetal calf serum added.

Equal volumes (0·05 ml) of the leucocyte suspension, tumour extract (when required) and undiluted pooled AB or autologous serum were mixed with TC 199 + 10% foetal calf serum, to give a final volume of 0·2 ml. Each of the mixtures was set up in duplicate. The tubes were randomized, coded and then incubated at 37°C for 30 min with constant mixing. In view of the short incubation time in this assay, antibiotics were not added to the culture medium.

Leucocyte adherence was then determined by introducing each mixture into the 2 chambers of a haemacytometer (Optical American Corporation). After a further 60 min incubation, the nucleated cells in a predetermined pattern of squares (16 squares/haemacytometer) were counted. The coverslips were then floated off and slides subjected to a gentle washing procedure; the remaining, adherent cells were then counted in the same squares.

The mean percentage adherence for each mixture was determined and a leucocyte adherence index calculated.

Leucocyte adherence index

$$= \frac{\text{Mean \% adherence with antigen}}{\text{Mean \% without antigen}}$$

RESULTS

The mean percentage adherence for each mixture and the leucocyte adherence indices for the control subjects, colorectal and other malignancies are shown in Tables I, II and III respectively.

The control subjects exhibited little or no reduced adherence in the presence of the tumour extract either in homologous AB serum (mean index 1·024 ± 0·0547 s.d.) or autologous serum (mean index 1·0421 ± 0·0866 s.d.). As there was no significant difference between the healthy subjects and the patients with benign disease, they have been treated as one group. No nonspecific or toxic effects of

TABLE I.—*Results of LAI Test in Control Patients*

| Patients | | | Mean % adherence | | | Leucocyte adherence index | |
|---|---|---|---|---|---|---|---|
| Age | Sex | Diagnosis | AB serum | AB serum +antigen | Auto serum +antigen | AB | Auto |
| 30 | M | Healthy | 53 | 49 | 47 | 0·92 | 0·9 |
| 61 | M | Healthy | 73 | 74 | 71 | 1·01 | 0·98 |
| 49 | M | Healthy | 57 | 64 | 63 | 1·12 | 1·1 |
| 69 | M | Healthy | 62 | 66 | 57 | 1·06 | 0·92 |
| 74 | M | Healthy | 46·5 | 47 | 54 | 1·01 | 1·16 |
| 45 | F | Healthy | 59 | 60 | 72 | 1·02 | 1·22 |
| 52 | M | Healthy | 75 | 71 | 79 | 0·95 | 1·05 |
| 53 | F | Healthy | 67·5 | 70·5 | 71·5 | 1·04 | 1·06 |
| 58 | F | Healthy | 70 | 71 | 67 | 1·01 | 0·96 |
| 61 | F | Healthy | 65·5 | 63·5 | 69 | 0·97 | 1·05 |
| 55 | F | Healthy | 71·5 | 71·3 | 76 | 0·99 | 1·06 |
| 59 | F | Healthy | 63 | 61 | 69 | 0·97 | 1·09 |
| 75 | M | Urethral stricture | 44 | 48 | 50 | 1·09 | 1·13 |
| 40 | M | Spontaneous pneumothorax | 69 | 71 | 67 | 1·03 | 0·97 |
| 71 | M | Angina | 56 | 64 | 57 | 1·14 | 1·07 |
| 45 | M | Gastric ulcer | 57 | 60 | 52 | 1·05 | 0·91 |
| 66 | M | Diverticulitis | 51 | 55 | 52 | 1·07 | 1·02 |
| 72 | M | Osteoarthritis | 59 | 58 | 68 | 1·02 | 1·15 |
| 50 | M | Fissure-in-Ano | 73 | 72·5 | 73 | 0·99 | 1·0 |

TABLE II.—*Results of LAI Test in Patients with Colorectal Cancer*

| Patients | | | | Mean % adherence | | | Leucocyte adherence index | |
|---|---|---|---|---|---|---|---|---|
| Age | Sex | Diagnosis | Stage* | AB serum | AB serum +antigen | Auto serum +antigen | AB | Auto |
| 45 | F | Ca colon | D | 73·5 | 36 | 31 | 0·49 | 0·43 |
| 52 | M | Ca colon | D | 62 | 69 | 65 | 1·1 | 1·03 |
| 68 | F | Ca colon | D | 58 | 74 | 43 | 1·27 | 0·74 |
| 76 | M | Ca rectum | D | 79 | 70 | 59 | 0·85 | 0·75 |
| 69 | M | Ca rectum | D | 46 | 48 | 67 | 1·04 | 1·45 |
| 67 | M | Ca rectum | C | 72 | 50 | 30 | 0·69 | 0·41 |
| 80 | F | Ca colon | C | 62 | 50 | 78 | 0·8 | 1·26 |
| 65 | M | Ca colon | C | 58 | 49 | 49 | 0·84 | 0·84 |
| 58 | F | Ca colon | D | 66 | 57 | 84 | 0·86 | 1·26 |
| 49 | M | Ca rectum | C | 69 | 54 | 72 | 0·78 | 1·05 |
| 69 | M | Ca rectum | D | 87 | 64 | 84 | 0·74 | 0·97 |
| 78 | M | Ca rectum | C | 75 | 74 | 84 | 0·99 | 1·12 |
| 53 | F | Ca colon | D | 80 | 77 | 81 | 0·96 | 1·01 |
| 70 | M | Ca colon | A | 55 | 58 | 57 | 1·05 | 1·03 |
| 68 | F | Ca colon | C | 57 | 42 | 60 | 0·74 | 1·05 |
| 77 | F | Ca rectum | B | 66 | 55 | 77 | 0·83 | 1·16 |
| 65 | M | Ca rectum | D | 48 | 42 | 38 | 0·87 | 0·81 |
| 73 | M | Ca rectum | D | 67 | 62 | 79 | 0·93 | 1·17 |
| 53 | M | Ca colon | B | 68 | 59 | 64 | 0·87 | 0·94 |
| 79 | F | Ca rectum | A | 41 | 44 | 44 | 1·07 | 1·07 |
| 67 | M | Ca colon | B | 70 | 77 | 67 | 1·1 | 0·96 |
| 54 | M | Ca colon | D | 64 | 54 | 65 | 0·78 | 1·01 |
| 67 | M | Ca colon | D | 53 | 41 | 43 | 0·76 | 0·81 |
| 71 | F | Ca colon | C | 52 | 55 | 62 | 1·05 | 1·19 |
| 68 | F | Ca colon | D | 83 | 73 | 84 | 0·88 | 1·02 |
| 62 | F | Ca rectum | A | 50 | 48 | 67 | 0·97 | 1·3 |
| 69 | M | Ca rectum | B | 69 | 56 | 65 | 0·81 | 0·94 |

* A = Confined to bowel.
B = Invading para-colonic tissues, nodes — ve.
C = Involvement of lymph nodes.
D = Distant metastases.

TABLE III.—*Results of LAI Test in Patients with Other Malignancies*

| Patients | | | | Mean % adherence | | | Leucocyte adherence index | |
|---|---|---|---|---|---|---|---|---|
| Age | Sex | Diagnosis | Stage* | AB serum | AB serum +antigen | Auto serum +antigen | AB | Auto |
| 66 | M | Seminoma | L | 72 | 68 | 65 | 1·07 | 1·05 |
| 75 | F | Ca breast | M | 71·5 | 59 | 83 | 0·8 | 1·1 |
| 62 | F | Ca thyroid | M | 49 | 47 | 64 | 0·96 | 1·3 |
| 57 | M | Ca lung | M | 86 | 64 | 51 | 0·74 | 0·59 |
| 58 | F | Rectal melanoma | M | 83 | 75 | 88 | 0·90 | 1·06 |
| 55 | F | Ca breast | M | 89 | 85 | 88 | 0·96 | 0·99 |
| 63 | M | Ca lung | L | 86 | 85 | 91 | 0·99 | 1·06 |
| 67 | M | Ca lung | L | 52 | 51 | 55 | 0·99 | 1·06 |
| 75 | M | Ca oesophagus | M | 52 | 52 | 59 | 1·0 | 1·13 |
| 83 | M | Ca stomach | M | 88 | 81 | 80 | 0·92 | 0·91 |
| 69 | F | Ca stomach | M | 77 | 76 | 79 | 0·99 | 1·03 |
| 74 | F | Ca cervix | L | 62·5 | 66 | 76 | 1·05 | 1·2 |
| 73 | M | Ca prostate | M | 77 | 77·5 | 81 | 1·0 | 1·05 |
| 73 | F | Ca stomach | M | 81 | 68 | 74 | 0·83 | 0·91 |
| 74 | F | Anal melanoma | M | 69 | 58 | 43 | 0·83 | 0·63 |
| 67 | F | Ca biliary tract | M | 73 | 57 | 45 | 0·8 | 0·61 |
| 75 | F | Ca stomach | M | 65 | 70 | 60 | 1·07 | 0·92 |
| 81 | F | Ovarian ca | M | 78·5 | 79 | 77·5 | 1·00 | 0·99 |
| 73 | F | Ca breast | L | 82 | 79 | 83 | 0·96 | 1·01 |
| 73 | M | Ca bladder | L | 68 | 65 | 78 | 0·96 | 1·15 |

* L = Localized; M = Metastatic.

the tumour extract were apparent in any of these tests.

Therefore, in this study, significantly reduced leucocyte adherence in the presence of the tumour extract is said to have occurred when the index falls below the 95% confidence limits (mean — 2 s.d.) calculated from all the control subject observations. The results obtained in AB serum are shown in Fig. 1 and those in autologous serum in Fig. 2.

In the patients with colorectal cancer of all stages 16/27 (59·25%) showed a significantly reduced index in homologous AB serum, whereas in autologous serum only 7 (26%) showed a significantly reduced index. Of the 20 patients with other malignancies, 6 (30%) showed a significantly reduced index in homologous AB serum and 3 (15%) in their autologous serum. The patients showing the sensitization in this group had metastatic cancer of the breast, lung, stomach, rectal melanoma (2) or biliary tracts. Patients without metastases were uniformly negative.

### DISCUSSION

This study has confirmed that the interaction between human leucocytes from patients with colorectal cancer and an extract of pooled colorectal tumours causes a reduced adherence of the cells to a glass surface. This reduced adherence does not appear to be due to toxicity of the tumour extract since adherence of the control subjects' leucocytes was not affected. Similarly, as 4 multiparous female control subjects showed no reduced adherence, presensitization to alloantigens seems unlikely. As the assay mixtures were incubated only for a short time, *in vitro* sensitization by histocompatibility antigens possibly present in the extracts can be excluded.

Unfortunately the LAI test as performed here appears to be of little use in the positive diagnosis of large bowel cancer since only 16 of the 27 patients studied exhibited significant reactivity to the colorectal tumour extract. A different type of extract may increase the sensitivity but it is of interest that similar proportions of patients are reported to show sensitization to tumour associated antigens in large bowel malignancy and other cancers by the leucocyte migration technique (Andersen *et al.*, 1970; Guillou and Giles, 1973).

No difference in reactivity in homologous AB serum was seen between patients whose tumours were locally confined (Dukes Stage A, B and C) and those with distant spread (Stage D). This clinical–pathological staging, although useful, probably does not truly represent the natural history of the disease. However, 4/4 with histologically de-differentiated tumours showed reactivity, 6/11 with moderately differentiated tumours and 6/12 with well differentiated growths.

A humoral inhibitory effect of autologous sera was present in 10/16 patients who showed reactivity when tested in homologous AB serum. This is analogous to the serum blocking factors reported using the lymphocyte cytotoxicity techniques (Hellström *et al.*, 1971*b*) and might represent one means by which the cellular immunological effects of sensitized leucocytes might be inhibited *in vivo* in the cancer patient. In our opinion, it is as an assay for serum blocking that the LAI test has its greatest potential use, particularly in sequential studies of post-excision patients, as the appearance of blocking factors is reported to herald recurrent growth.

The LAI test is claimed to be a rapid and simple *in vitro* method of detecting sensitization to tumour associated antigens (Maluish and Halliday, 1974). In our experience the tedious visual cell counting and technical dexterity necessary for consistent results might limit its usefulness. The modification of the test by a radioisotope technique, recently described by Peirce and Devald (1974) in animals, could overcome these difficulties if successfully applied to man.

In this study the tumour antigen preparation was extracted with perchloric acid, an established method for preparing carcinoembryonic antigen (CEA). Indeed, our extract has been shown to contain CEA, among other antigens, by the use of heterologous antiserum and a purified CEA preparation (Medical Research Council, CEA C73/601). The adherence inhibition was specific for patients with tumours but not organ specific. The other carcinomata which were reactive were of types which can produce elevated serum CEA levels (Pusztaszeri and Mach, 1972). It is of interest to speculate on its immunogenic role, although Lejlenyi, Freedman and Gold (1971) found no evidence for a cell mediated immune reaction to purified CEA using a lymphocyte transformation technique. Hellström, Hellström and Shepard (1970) demonstrated that lymphocytes from patients with carcinoma of the colon, which reacted specifically with colon cancer cells *in vitro*, were also cytotoxic to foetal gut and liver epithelial cells; also, a delayed skin reaction to extracts of foetal human gut tissue containing CEA was found in patients with carcinoma of the colon and rectum (Hollinshead *et al.*, 1970), however purified CEA elicited no skin reaction in the one patient tested. The relationship between embryonic macromolecules found in tumour tissue and antigens involved in anti-tumour immunity still remain to be resolved.

We thank the surgeons of Leeds (St James's) University Hospital for allowing us to study patients under their care and also the Medical Research Council for their gift of CEA.

### REFERENCES

ANDERSEN, V., BJERRUM, O., BENDIXEN, G., SCHIØDT, T. & DISSING, I. (1970) Effect of Autologous Mammary Tumour Extracts on Human Leukocyte Migration *in vitro*. *Int. J. Cancer*, **5**, 357.

FREED, D. L. G. & TAYLOR, G. (1972) Carcinoembryonic Antigen in Faeces. *Br. med. J.*, i, 85.

GUILLOU, P. J. & GILES, G. R. (1973) Inhibition of Leucocyte Migration by Tumour Associated Antigens of Colon and Rectum. *Gut*, **14**, 733.

HALLIDAY, W. J., MALUISH, A. & ISBISTER, W. H. (1974*a*) Detection of Anti-tumour Cell-mediated Immunity and Serum Blocking Factors in Cancer Patients by the Leucocyte Adherence Inhibition Test. *Br. J. Cancer*, **29**, 31.

HALLIDAY, W. J., MALUISH, A. & ISBISTER, W. H. (1974*b*) Blocking and Unblocking of Cell-mediated Immunity in Mice as detected by the L.A.I. Test. *Cell. Immun.*, **10**, 467.

HELLSTRÖM, I., HELLSTRÖM, K. E. & SHEPARD, T. H. (1970) Cell-mediated Immunity against Antigens Common to Human Colonic Carcinomas and Fetal Gut Epithelium. *Cancer, N.Y.*, **6**, 346.

HELLSTRÖM, I. E., HELLSTRÖM, K. E., SJÖGREN, H. O. & WARNER, G. A. (1971*a*) Demonstration of Cell Mediated Immunity to Human Neoplasms of Various Histological Types. *Int. J. Cancer*, **7**, 1.

HELLSTRÖM, I., SJÖGREN, H. O., WARNER, G. & HELLSTRÖM, K. E. (1971*b*) Blocking of Cell-mediated Tumour Immunity by Sera from Patients with growing Neoplasms. *Int. J. Cancer*, **7**, 226.

HOLLINSHEAD, A., GLEW, D., BUNNAG, B., GOLD, P. & HERBERMAN, R. (1970) Skin Reactive Soluble Antigen from Intestinal Cancer-cell-membranes and Relationship to Carcinoembryonic Antigens. *Lancet*, i, 1191.

LEJLENYI, M. C., FREEDMAN, S. O. & GOLD, P. (1971) Response of Lymphocytes from Patients with Gastro-intestinal Cancer to CEA of the Human Digestive System. *Cancer, N.Y.*, **28**, 115.

MALUISH, A. & HALLIDAY, W. G. (1974) Cell Mediated Immunity and Specific Serum Factors in Human Cancer: The LAI Test. *J. natn. Cancer Inst.*, **52**, 1415.

PIERCE, G. E. & DEVALD, B. L. (1974) Leucocyte Adherence Inhibition as Measured by a Radioisotope Technique for Detection of Cell-mediated Tumour Immunity. *Int. J. Cancer*, **14**, 833.

PUSZTASZERI, G. & MACH, J. P. (1972) Tumour Immunity. Carcinoembryonic Antigen in Non-digestive Cancerous and Normal Tissues. *Immunochemistry*, **10**, 197.

VANKY, F., STJERNSWARD, G., KLEIN, G. & NILSONNE, V. (1971) Serum Mediated Inhibition of Lymphocyte Stimulation by Autochthonous Human Tumors. *J. natn. Cancer Inst.*, **47**, 95.